# On the Applicability of Camera Lens Protectors in Emergency Luminescence Dosimetry

**DOI:** 10.3390/ma15010193

**Published:** 2021-12-28

**Authors:** Renata Majgier, Kordian Chamerski, Arkadiusz Mandowski

**Affiliations:** Department of Experimental and Applied Physics, Faculty of Science and Technology, Jan Dlugosz University, ul. Armii Krajowej 13/15, 42-200 Czestochowa, Poland; kordian.chamerski@ujd.edu.pl (K.C.); a.mandowski@ujd.edu.pl (A.M.)

**Keywords:** camera lens protectors, glass, emergency dosimetry, luminescence dosimetry, ionizing radiation

## Abstract

In this work, the optically stimulated luminescence (OSL) properties of camera lens protectors and their potential use in emergency dosimetry were investigated. Camera lens protectors can be attached to mobile phones, which are commonly carried by individuals and may be useful in estimating an emergency dose. The presented results confirm the great potential of this type of glass material for dose determination. The glass protectors exhibit advantageous properties, such as linear dose dependence in the range of at least 0.6–10 Gy, minimum detectable dose at the level of tens of mGy, and good measurement repeatability for samples of the same type. Significant fading during the first day after exposure is an undesirable feature of tested glass. Nevertheless, the application of the correction for fading shows promising results in the dose recovery process.

## 1. Introduction

The interest in emergency dosimetry results from the growing role of nuclear power plants in electricity generation and the use of ionizing radiation in medicine, science, and industry, which increases the probability of situations where the population may be exposed to radiation, and large areas may be contaminated by radiation. In dosimetry practice, many measurement methods are used, including those that use luminescent phenomena-thermoluminescence (TL) and optically stimulated luminescence (OSL). The TL and OSL usually occur in crystalline dielectric materials with a wide energy band gap, but also in materials that are not crystalline in their nature, such as glass and glass–ceramics. The all-optical nature of the OSL method indicates its advantage over TL and has caused an increased interest in OSL. Detectors commonly used in the OSL method in individual and environmental dosimetry are aluminum oxide doped with carbon (Al_2_O_3_:C) and beryllium oxide (BeO). In situations where commercial detectors are not available, other phosphors located in the place of exposure can be used as an emergency detector. New materials useful for OSL emergency dosimetry are being intensively researched in the world. Among the materials tested for this purpose, there are elements of mobile phones: electronic components such as resistors, inductors, and integrated circuits [1,2,3,4,5], as well as display glass [6,7] and protective glass [8,9,10]. Similar studies of electronic components [11,12] and display glass [13,14,15,16,17] and protectors [18,19] were also carried out using the TL method. External glass elements (protective glass) placed on the phone have the advantage over internal electronic components in that there is no need to destroy the phone in order to take a sample. Protectors were tested using both TL and OSL methods, wherein TL studies showed a high background of the non-irradiated sample [19], which may make dose determination difficult, while the background of OSL signals were negligible [9]. Sholom et al. [8,9] proposed a non-destructive readout of back protective glasses from modern smartphones for possible application in post-exposure radiation triage following a radiological accident. Their results showed strong radiation-induced OSL signals with significant variability in sensitivity among the different phones, linear dose–response relationships for all phones in the range of 0–2.7 Gy [9] or 0.1–8 Gy [10], minimum detectable dose in the order of Gy [8] or mGy [9], and fast fading during the first day after irradiation [8,9,10]. Performed dose recovery tests conducted on two phones in daily use showed that the deviation of the fading-corrected OSL doses from corresponding nominal values was within 25% [9]. The above-mentioned studies show the high potential of protective glass in emergency dosimetry and indicate the need for further research in this direction.

In this work, several types of protective glass for the camera lens were tested. The selected protectors have a round shape, adapted to the size of the lens of a given phone model. The protective glass for the camera lens can also be used on other devices, e.g., laptops. The glass samples can be easily taken from the devices and directly inserted into the OSL reader, without the need for additional preparation. Additionally, if necessary, the glass samples can be protected from the light by the camera cover, which is used to ensure privacy and security. The aim of the work is to investigate the dosimetric properties of various commercially available camera lens glass protectors, showing the potential of the tested materials for use in emergency dosimetry.

## 2. Materials and Methods

**Figure 1:**
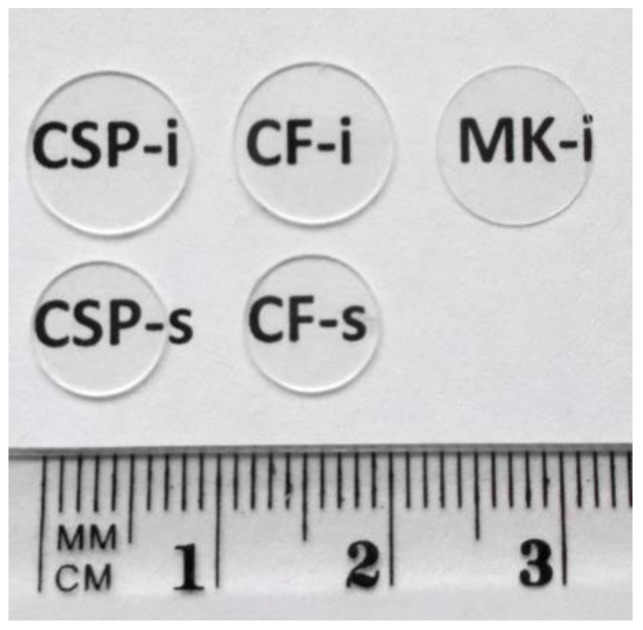
Photos of tested camera lens glass protectors with markings.

The study was carried out on various types of protective glass for the camera lenses. Five different types of toughened glass were tested. A detailed description of glass samples with parameters is presented in Table 1. All samples had a round shape and a diameter adapted to the dimensions of the phone camera for which they were intended. The appearance of the studied protective glass is shown in Figure 1. The samples were tested without additional preparation. The OSL measurements were carried out on a series of samples of each type; the given sample was measured once, except for reusability and sensitivity changes measurements where one sample was measured several times. Laboratory irradiations were made using ^90^Sr/^90^Y beta sources located in sheltered irradiators: one with dose rate of about 0.86 mGy·s^−1^ at the sample position (irradiator type UN-85, Institute of Nuclear Physics PAN in Krakow, Poland) and the second with dose rate of about 59.2 mGy·s^−1^ at the sample position (irradiator Daybreak type 801E). The appropriate dose is delivered by irradiating the sample for a specified period of time, with only one sample at a time in the irradiator. The exposure time is set in the irradiator, ensuring a reproducible dose value. The lower dose rate source was used to measure the dose–response dependence in the range of 0.01–0.5 Gy and to deliver a test dose of 0.06 Gy, while the higher dose rate source was used for all other measurements.

The OSL measurements were performed using a Helios OSL reader (manufactured by Zero-Rad, Czestochowa, Poland) [20], working in continuous wave OSL (CW-OSL) mode, where the sample is illuminated with light of constant intensity during the measurement. The stimulation was carried out using fifteen green LEDs (peak at 520–532 nm) with optical lenses, operating with a current set at 80 mA. Additional stimulation filters (Schott filters GG495 and OG515, Mainz, Germany) were used to cut off the shortwave light component below 500 nm. Detection of the OSL signal was performed in the range of 300–380 nm using Schott UG11 filters and a H7360 photocounter (Hamamatsu Photonics, Hamamatsu, Japan) with a quartz window and counter electronics with a computer interface.

## 3. Results and Discussion

### 3.1. Basic OSL Properties: Sample Background, CW-OSL Decay, Repeatability of the OSL Signal

**Figure 2:**
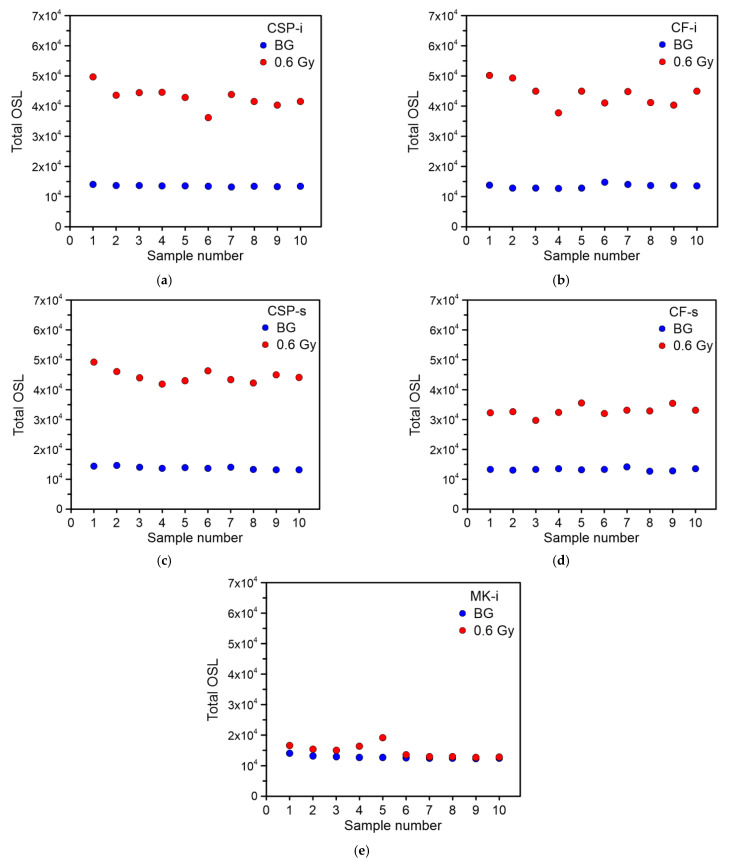
The sum of the OSL signal counts (0–60 s) for ten non-irradiated samples (background) and ten (the same) samples irradiated with a dose of 0.6 Gy: (**a**) CSP-i, (**b**) CF-i, (**c**) CSP-s, (**d**) CF-s, (**e**) MK-i.

**Figure 3:**
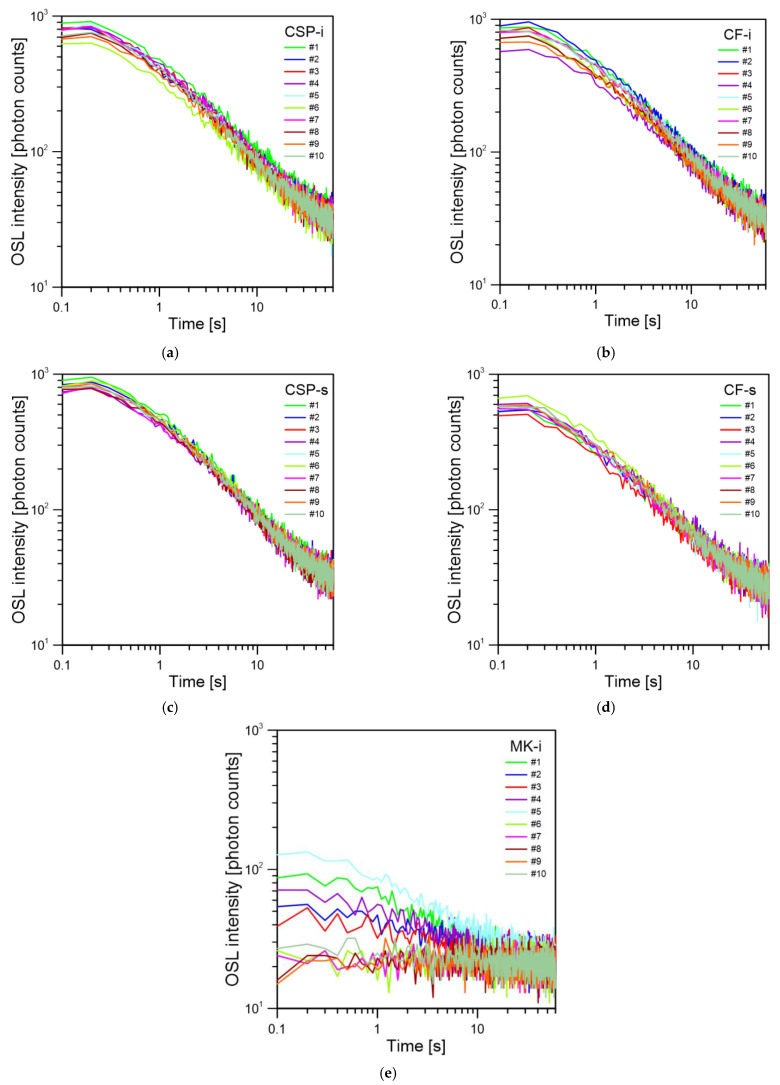
The CW-OSL decays for ten samples irradiated with a dose of 0.6 Gy and measured in the same conditions: (**a**) CSP-i, (**b**) CF-i, (**c**) CSP-s, (**d**) CF-s, (**e**) MK-i.

**Figure 4:**
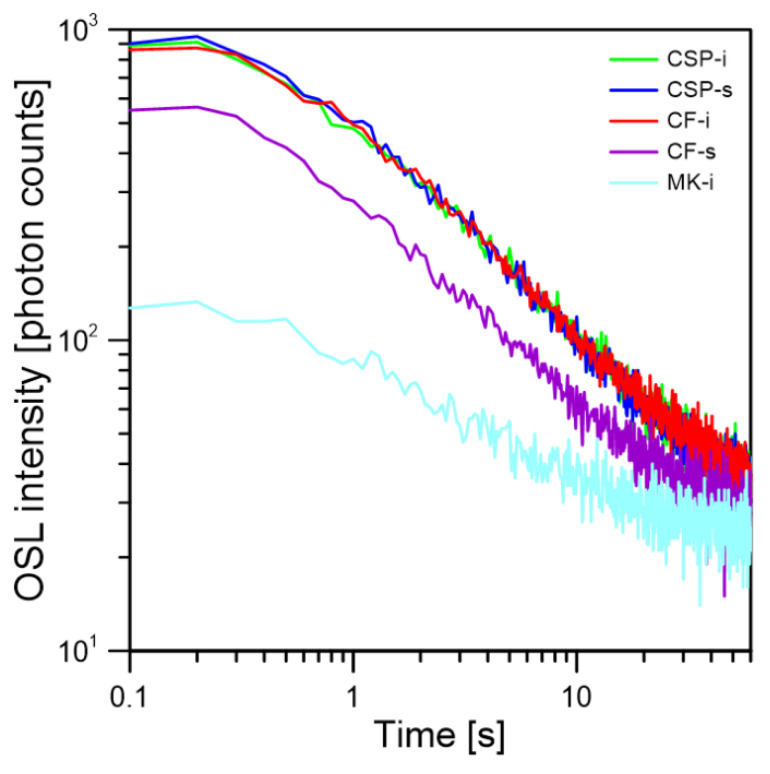
Comparison of the OSL decays (with the highest intensity among a given type) for the five types of samples tested, namely CSP-i, CF-i, CSP-s, CF-s, and MK-i, obtained after irradiation with a dose of 0.6 Gy.

The first step in the research was to check whether the glass samples exhibit a natural background (BG). For this purpose, ten samples of each type were measured without any irradiation. None of the measured samples showed an increased OSL signal, and the BG was on a similar level for a given type of glass. The standard deviation of the total background (σ BG) signal measured in relation to the average BG signal ranged from 1.6% for the CSP-i type samples to 4.9% for the CF-t samples. The other glass types had a deviation of 3.1%, 3.7%, and 3.9% for CF-s, CSP-s, and MK-i, respectively. The sum of the total BG signal for all samples of all tested glasses is shown in Figure 2a–e. It was compared also to the total OSL signal (the sum of OSL counts in the entire time range, 0–60 s) for the same samples irradiated with a dose of 0.6 Gy. For most of the samples, the radiation induced signal was significantly higher than the BG level, except for the MK-i samples. The CW-OSL decays measured on ten samples for all types of glass is shown in Figure 3a–e. Each of the tested glass types was characterized by a similar decay shape in their group with a comparable intensity. The standard deviation for the irradiated samples of the same type was greater than for the background—the standard deviation to mean ratios were 4.93%, 5.04%, 8.10%, 8.95%, and 14.65% for CSP-s, CF-s, CSP-i, CF-i, and MK-i, respectively. The highest deviation between individual OSL signals was observed for the MK-i samples. The MK-i samples also showed the lowest OSL signal among all tested types of glass, which can be seen in Figure 4, where the selected decays with the highest intensity for different types of glass were compared; these were, respectively, #1 for CSP-i, #1 for CSP-s, #1 for CF-i, #5 for CF-s, and #5 for MK-I, as seen in Figure 3. The MK-i samples were the least sensitive to radiation and showed greater differences in the results obtained under the same measurement conditions, which may indicate that this type of glass has worse dosimetric properties than the others. The three types of glass, namely CSP-i, CSP-s, and CF-i, showed very similar sensitivity and high measurement repeatability, which proved their dosimetry potential.

### 3.2. Reusability and Sensitivity Changes

**Figure 5:**
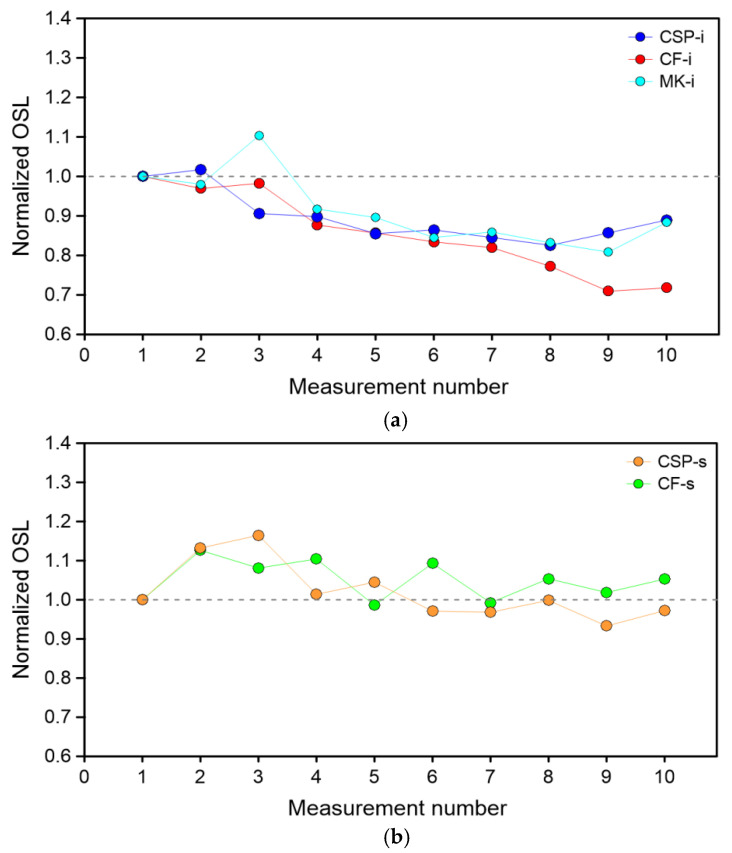
Sensitivity changes with the subsequent OSL measurement for the samples: (**a**) CSP-i, CF-i, MK-i, (**b**) CSP-s, CF-s.

Reusability is the important dosimetric property and indicates efficiency of dosimetric material during the multiple irradiation and OSL readout process. Some emergency dosimetry materials show sensitivity changes during multiple measurements [4,21,22]. As demonstrated by Chandler et al. [10], the changes in sensitivity may be negligible for some types of protective glass. To study the reusability and sensitivity changes of camera protective glass, the following procedure was used: a selected sample of a given type was irradiated with a dose of 0.6 Gy, and then after a short period of storage (60 s), the OSL was acquired for 60 s. After the OSL readout, the sample was bleached using blue light (150 s) to completely remove the remaining signal from the sample. This procedure was repeated 10 times. The relative OSL readout, normalized to the value obtained for the first measurement, was plotted as a function of the measurement number and is shown in Figure 5. Obvious sensitivity changes were observed in cases of all types of tested glass, and a clear downward trend was observed for CF-i, CSP-I, and MK-i. In the case of CF-i glass, the sensitivity dropped to 70% of the original value on the tenth measurement. In the case of the other two types mentioned, the decrease was smaller, down to about 80% of the original value at the eighth measurement, with no further decrease observed. On the other hand, in the case of CSP-s and CF-s samples, a significant decrease in the signal with successive measurement was not observed, and the relationship was irregular.

**Figure 6:**
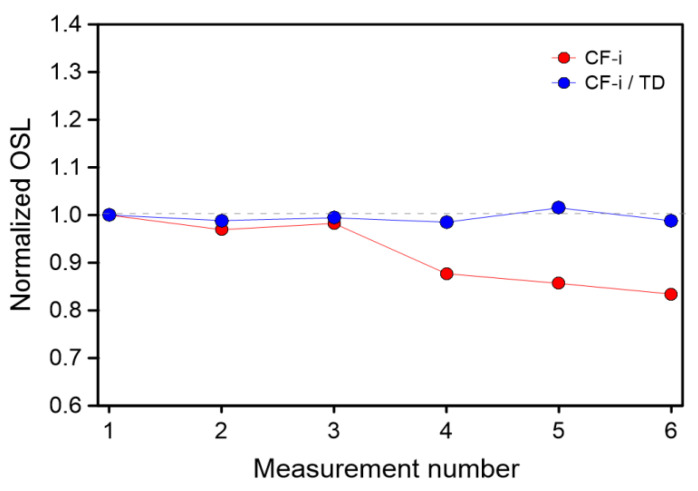
Comparison of sensitivity changes for CF-i sample without and with the test dose correction procedure.

Changes in sensitivity for the tested samples indicated the necessity to use the method of correction of these changes, such as test dose corrections, to demonstrate the applicability of a protocol-type dose determination procedure, e.g., SAR [23]. In the case of other glass protectors described in the literature, the authors do not indicate the necessity of a test dose correction [10]. However, in the case under examination, during multiple readout of one sample, a correction procedure should be followed. To investigate the possibility for correcting the sensitivity changes, the following procedure of test dose (TD) was applied; the sample was irradiated with a dose of 0.6 Gy and then stored for 60 s before the OSL signals were acquired (readout during 60 s). After the readout, additional blue light bleaching for 150 s was applied to completely reset the signal from the sample. Directly after bleaching, a TD of 0.06 Gy was administered to the sample. After storage, the OSL signal was measured (60 s), and the sample was bleached again (150 s). This cycle was repeated six times for each sample. The result of the test dose correction procedure for the CF-i sample is shown in Figure 6. The CF-i sample was chosen as the representative sample because the changes in sensitivity for it were the highest. As can be seen, these changes can easily be corrected using a test dose and such a correction can be used in dosimetric practice while measuring camera glass protectors.

### 3.3. Dose–Response and Theoretical Detection Limit

A desirable feature of the OSL detector is linear dose dependence in a broad dose range. This means that the luminescence response, which is the result of recombination of charges in the material after light stimulation, increases linearly with the received dose. In practice, situations where linearity occurs over the entire dose range (up to saturation) are rare, and in the case of lower or higher doses, sublinear or superlinear dose dependencies take places. For the purposes of emergency dosimetry, it is important that the material has linearity in the range of 0.5–10 Gy [24], as then it is much easier to determine the dose using the calibration curve approach.

**Figure 7:**
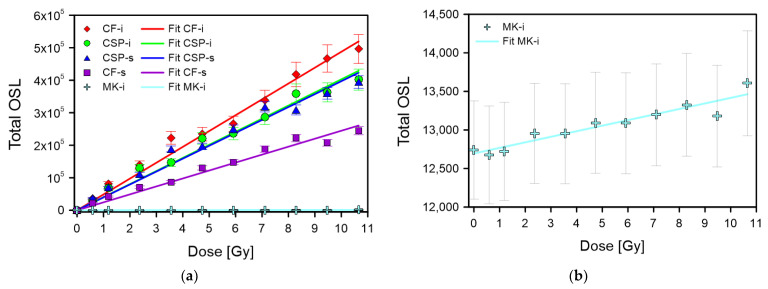
Dose–response characteristics measured in the range of 0.6–10 Gy with a linear fit using the y = ax function: (**a**) for all sample types, (**b**) for MK-i.

**Figure 8:**
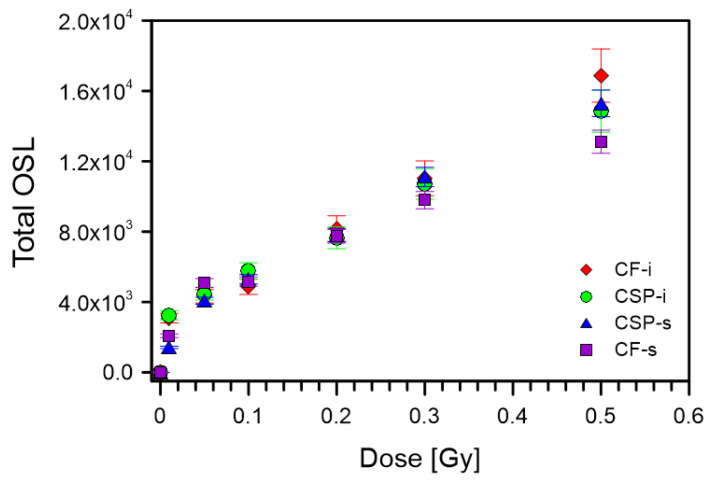
Dose–response characteristics measured in the range of 0.01–0.5 Gy for CSP-i, CF-i, CSP-s, CF-s samples.

Dose–response characteristics were studied using two different irradiation sources, one with a higher dose rate (59.2 mGy·s^−1^) for measurements in the range of 0.6–10 Gy, and the other with a lower dose rate (0.86 mGy·s^−1^) for measurements from 0.01 Gy to 0.5 Gy. The results obtained for higher doses of 0.6–10 Gy are shown in Figure 7a,b. Over the entire range of studied doses, a linear relationship was observed. After subtracting the background, it was possible to fit a line with the equation y = ax to the measuring points, which allowed the specific value to be determined of the counts per dose. The dose–response dependence for the lower doses is shown in Figure 8. The dependence for MK-i glass is not shown due to the very small signal. The relationship is clearly sublinear, especially below 0.1 Gy in all the cases shown.

Assuming a linear dose–response relationship in the range of 0.6–10 Gy, the minimum detectable dose (MDD) was estimated as the ratio of three times the standard deviation of the background (3σ BG) to the sample specific sensitivity (c_spec_). The estimated MDDs were 36 ± 1 mGy, 16 ± 1 mGy, 38 ± 1 mGy, and 50 ± 1 mGy for CF-i, CSP-i, CSP-s, and CF-s, respectively. The value of the MDD depended on the time after irradiation due to strong fading occurring in the studied material (see Section 3.4). The MDDs calculated one hour and one day after exposure are shown in Table 2. The increase in MDDs from tens to hundreds of mGy was evident. In the case of MK-i, an increasing dose relationship was also noticeable; however, the number of counts for subsequent doses was rather small compared to the background level. Figure 7b shows the dose–response relationship for the MK-i samples, with dose zero showing the mean background level measured for ten samples (see Section 3.1), and the counts for subsequent doses are shown without background subtraction. As can be seen, the noticeable increase in the signal started above 2 Gy. The MDD estimated from the slope of this dose relationship and BG standard deviation for this type of sample was 2.6 ± 0.1 Gy. The obtained MDD value and the dose–response relationship confirmed significantly worse properties of the MK-i type glass compared to the other samples.

### 3.4. Fading in Darkness and in Daylight

**Figure 9:**
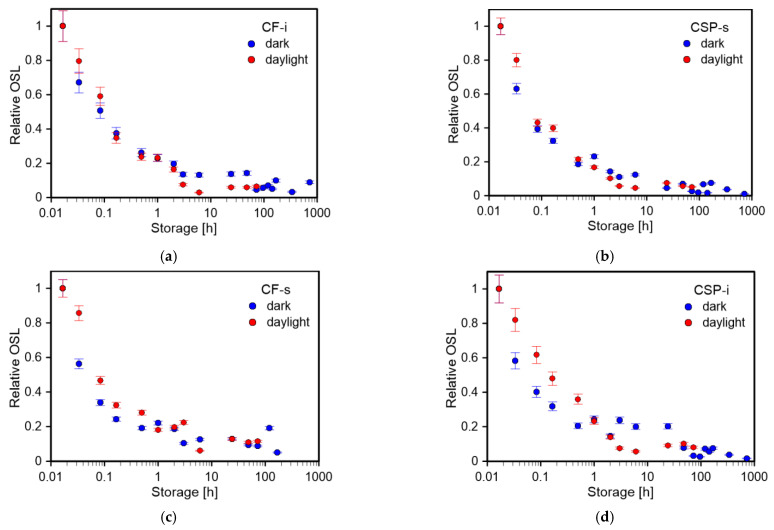
Fading characteristics-relative OSL (normalized to the readout immediately after irradiation) as a function of storage time after irradiation of 0.6 Gy. The graph compares fading after dark storage and daylight storage for (**a**) CF-i, (**b**) CSP-s, (**c**) CF-s, and (**d**) CSP-i.

The fading characteristics were investigated for samples stored in the dark as well as with access to daylight. The samples were irradiated with a dose of 0.6 Gy and then stored for some time in a light-proof box (fading in darkness) or in transparent foil (fading in daylight) until the OSL signal was read. Figure 9a–d shows the relative OSL as a function of storage time for CF-i, CSP-s, CF-s, and CSP-i samples, respectively. The figure compares two relationships, signal loss during daylight and dark storage. Measurement points for successive storage times were normalized to the readout immediately after irradiation that was carried out after transferring the sample in the dark (using red light) or in daylight, respectively. It should be noted that the corresponding normalization readouts immediately after irradiation in dark or in light were at a similar level. As can be seen in Figure 9, in all cases, both for dark storage and daylight storage, a significant decrease in signal was observed within one day after irradiation. After this time, the signal dropped slightly and remained at a level of a few percent of the initial value. One day after irradiation, the signal was at 6–13% of the initial value for samples stored in daylight and at 13–20% for samples stored in the dark. The existence of such significant fading is of importance for dosimetric applications. It is necessary to use fading correction in the dose determination procedure. This procedure was used to retrieve the dose information described in the next section. Similar rapid fading on the first day and the need for dose correction due to fading was reported by other researchers for various protectors [9,10].

### 3.5. Dose Recovery Test

The dose recovery test was performed on four types of glass samples (excluding MK-i), showing a good dosimetric potential. The samples were irradiated with doses of 0.258 Gy, 1.77 Gy, and 2.95 Gy, respectively. The selected doses represented three levels of risk in emergency dosimetry (radiation dose triage levels): <1 Gy low, 1–2 Gy medium, and >2 Gy high [24]. After irradiation, the samples were stored in a non-fully light-tight package. The OSL readout was performed one day after irradiation. The dose estimation was as follows: the sample-specific background was subtracted from the measured OSL signal, the dose was then read from the appropriate calibration curve (dose–response) based on the obtained OSL intensity, and finally the dose was corrected for the fading factor. Due to the similar fading characteristics one day after irradiation for all samples, as well as mixed dark–daylight storage conditions, it was decided to set a common fading correction factor. The average fading correction factor for one day after irradiation was determined to be 0.140 ± 0.028. This factor was used for dose corrections in all cases. The results of the performed dose recovery test are shown in Table 3. The recovered doses were within 4–58% of the nominal value. The smallest deviation in the case of low and high doses was demonstrated by the CSP-s sample, and in the case of medium doses by the CF-i. The CF-i sample, however, showed the highest deviation at low doses. The CF-s sample had the largest deviation for medium and low doses. Nine of the twelve dose recovery samples gave the dose correctly within the uncertainty limits. For the majority of samples (ten out of twelve) the dose could be correctly assigned to a category; in the other two cases (CSP-i, CF-s for dose of 1.77 Gy) the estimated dose might be assigned to a higher level or an intermediate level. The average correction factor was used as the optimal method of dose determination, after numerous calculations also with averaged specific correction factors only for a given type of glass. The application of sample type specific correction factors may result in an improvement in the determination of the nominal dose, e.g., for the CF-s sample for the nominal doses of 1.77 Gy and 2.95 Gy, smaller values of 2.35 ± 0.14 Gy and 3.21 ± 0.19 Gy, respectively, were obtained. However, in cases where the spread between the value of fading in darkness and lightness of specific samples is higher, as is the case for samples other than CF-s, applying averaged coefficients for all samples gives more accurate results. For the purpose of segregation and determination of the risk level, the method seems to be sufficient.

## 4. Conclusions

The studies showed that some camera lens glass protectors have the potential for use as emergency detector materials for accidental dose assessment. The glass under study can be easily removed from a phone or other device not needing additional processing to measure the dose. Five different types of glass were tested, four of which showed good dosimetric properties. These properties include linear dose dependence in the range of at least 0.6–10 Gy, the minimum detectable dose at the level of tens of mGy, good measurement repeatability for samples of the same type, the possibility of correction of sensitivity changes with the test dose. An undesirable feature of the tested materials is fading, which is significant during the first day after exposure. However, the dose recovery test showed that doses close to the nominal values can be obtained by applying the fading correction. The doses determined in the estimation process differed from the nominal ones from 4% to 58%. For half of the dose recovery trials, the deviation from the nominal dose was less than 20%, which is considered as a quite good result for emergency dosimetry. The first attempts to estimate the dose with camera lens glass protectors carried out in laboratory conditions give good hope for the application of the materials in dosimetric practice. Moreover, the lens protector seems to be a much better and more reliable detector than the main display protector, which is susceptible to bleaching under the influence of a much stronger light of the display. To apply camera lens glass protectors in more realistic emergency situations, further detailed research on these materials are needed.

## Figures and Tables

**Table 1:** List and parameters of tested camera lens glass protectors.

Designation	Manufacturer	Phone Model	Thickness [mm]	Diameter [mm]	Hardness
CSP-i	Camera Screen Protector	iPhone 12	0.3	9.5	9 H
CF-i	Camera Film	iPhone 12	0.3	9.5	9 H
CSP-s	Camera Screen Protector	Samsung Galaxy A32	0.3	8	9 H
CF-s	Camera Film	Samsung Galaxy A32	0.3	8	9 H
MK-i	3 mk Protection	iPhone 12	0.2	9.3	7 H

**Table 2:** MDDs values of camera lens glass protectors determined for various times after exposure to radiation.

Sample	MDD (mGy)		
	Immediately after Irradiation	1 h after Irradiation	1 Day after Irradiation
CSP-i	16 ± 1	73 ± 9	115 ± 26
CF-i	36 ± 1	186 ± 21	292 ± 39
CSP-s	38 ± 1	173 ± 21	272 ± 63
CF-s	50 ± 1	229 ± 28	360 ± 82

**Table 3:** Results of the dose recovery test using camera lens glass protectors.

Sample	Nominal Dose (Gy)	Recovered Dose (Gy)	Deviation from the Nominal Dose (%)
CSP-i	0.26	0.19 ± 0.13	−27%
CSP-s		0.27 ± 0.12	5%
CF-i		0.38 ± 0.14	49%
CF-s		0.16 ± 0.15	−40%
CSP-i	1.77	2.18 ± 0.48	23%
CSP-s		1.43 ± 0.30	−19%
CF-i		1.70 ± 0.38	−4%
CF-s		2.79 ± 0.59	58%
CSP-i	2.95	2.48 ± 0.54	−16%
CSP-s		2.79 ± 0.59	−5%
CF-i		2.61 ± 0.58	−12%
CF-s		3.81 ± 0.80	29%

## Data Availability

Data are available upon request from the corresponding authors.
